# Uric acid/superoxide dismutase can predict progression of gestational hypertension to preeclampsia

**DOI:** 10.3389/fcvm.2023.1148376

**Published:** 2023-03-30

**Authors:** Lin Yun, Xiaoqian Yu, Rui Xu

**Affiliations:** ^1^ Department of Cardiology, Jinan Central Hospital, Shandong University, Jinan, China; ^2^ Department of Medicine, Jinan Maternity and Child Care Hospital, Jinan, China; ^3^ Department of Cardiology, Central Hospital Affiliated to Shandong First Medical University, Jinan, China

**Keywords:** gestation hypertension, preeclampsia, uric acid, superoxide dismutase, predictive model

## Abstract

**Introduction:**

Preeclampsia (PE), at early onset, is likely to be diagnosed as gestational hypertension (GH). Some cases of GH rapidly progress to PE within a short period of time, increasing the mortality rate of pregnant women and adverse events in neonates during the peripartum period. Oxidative stress participates in the occurrence and progression of PE. However, it is unknown whether the progression of GH to PE can be predicted.

**Methods:**

A total of 1548 patients diagnosed with PE (649 cases) or GH (899 cases) from January 2016 to June 2022 were selected as the study subjects. The 1548 patients were randomly divided into the training set (1083 cases) and the validation set (465 cases) in a 7:3 ratio. General and clinical data were collected to construct a risk factor prediction model for PE.

**Results:**

We found that (1) Systolic blood pressure (SBP), and uric acid (UA)/ superoxide dismutase (SOD) were the risk factors for the progression of GH to PE; (2) A nomogram was constructed from the prediction model, and the area under the curve (AUC) was 0.95, with a sensitivity of 87.4%, a specificity of 92.8%; (3) Build a model simplified scoring system. PE was most strongly predicted by UA/SOD (100 points), SBP (29 points), and serum potassium (19 points). The AUC was 0.92, with a sensitivity of 91.0%, a specificity of 81.7%. The clinical decision analysis curve shows that the model exhibits positive benefits when the threshold probability is at 0.01–0.91.

**Conclusion:**

These findings show that UA/SOD can predict progression of GH to PE.

## Introduction

Hypertensive disorders of pregnancy (HDP) are among the main causes of mortality in pregnant women and peripartum infants. The global incidence of HDP is 7%–12%, and it accounts for 14% of global maternal deaths (1). Preeclampsia (PE), an important component of HDP, has an incidence of 3%–5% (2). PE is one of the main causes of maternal and child mortality (3). Gestational hypertension (GH) in HDP and PE are the main factors that increases the risk of adverse obstetric outcomes. Around 10%–20% of GH patients will progress to PE (4). Making the diagnosis of PE has become more complicated since the American College of Obstetricians and Gynecologists and International Society for the Study of Hypertension in Pregnancy updated their guidelines (5, 6); it now includes heart, brain, liver, and kidney damage as well as hematologic, digestive, and nervous system abnormalities. In clinical practice, it is usually difficult to block disease progression after the patient has been diagnosed with PE. Some PE patients have severe disease at the time of diagnosis. They had to terminate the pregnancy within a short period of time. The early identification of women at high risk of developing PE and timely intervention can help improve maternal and infant outcomes.

PE is an idiopathic pregnancy disorder with a complex pathogenesis and insidious onset. Compared with GH, PE involves more pathogeneses (7). Currently, widely recognized mechanisms include the two-stage placental hypothesis and oxidative stress (8, 9). As the occurrence and progression of PE are complex, its pathogenesis is still not elucidated in relevant studies. Difficulties and challenges persist in the early prediction and treatment of PE in clinical practice. Oxidative stress plays an indispensable role in the occurrence and progression of PE. Under normal circumstances, the body's oxidant and antioxidant systems are in equilibrium. In pregnant women, a genetic or immune disorder can cause placental vascular remodeling disorder, leading to a reduction in placental blood flow. Placental hypoxia can produce a large amount of oxygen free radicals, but antioxidant capacity is insufficient at this time and the equilibrium is disrupted. This causes vascular endothelial cell damage and further promotes PE occurrence and progression (10). Uric acid (UA) is the final product in purine metabolism, an important oxidant *in vivo*, and mainly eliminated by the kidneys (11). As glomerular filtration rate and blood dilution increases, blood UA level increases during pregnancy (12). A case control study on PE vs. healthy pregnant women reported that serum UA level is significantly increased in pregnant women with PE and every unit increase in UA increases the risk of PE development by 1.98 times (13). Moreover, superoxide dismutase (SOD) is an important antioxidant that protects tissue from oxidative damage and can protect blood vessels from ROS damage and maintain vascular function (14). SOD is significantly decreased in endothelial cell injury diseases (15, 16). A meta-analysis of 2,953 PE pregnant women and 3,621 healthy pregnant women found that a total of 26 studies described the SOD level of 739 PE pregnant women and 906 healthy pregnant women. It was reported that SOD and PE were significantly correlated with an effect size of 1.27 (95% CI: 1.774–0.768, *P* < 0.01) (17). UA and SOD are markers of oxidative stress that are cheap, easily obtained, and can reflect *in vivo* oxidant and antioxidant levels.

However, it remains unknown whether UA/SOD can predict the progression of GH to PE. In this study, a real-world big data analysis was performed from the perspective of oxidative stress to construct and validate a clinical prediction model of GH progression to PE and examine whether the oxidative stress marker UA/SOD can effectively predict PE. Our findings will provide a basis for the early discovery and identification of women at high risk of developing PE in clinical practice.

## Methods

### Data collection

A total of 1,548 patients diagnosed with PE (649 cases) or GH (899 cases) from January 2016 to June 2022 were selected as the study subjects. The 1,548 patients were randomly divided into the training set (1,083 cases) and the validation set (465 cases) in a 7:3 ratio. General and clinical data (within 1 week before the end of pregnancy) were collected which included age, heart rate (HR), blood pressure (BP), height, weight, and body mass index (BMI), as well as some indicators related to oxidative stress like blood urea nitrogen (BUN), total bilirubin (TBIL), total bile acid (TBA), free fatty acid (FFA), direct bilirubin (DBIL), alkaline phosphatase (ALP), serum creatinine (Scr), cystatin C, glucose (GLU), SOD, Na+ and K+. The study protocol was approved by the Ethics Committee of Jinan Maternal and Child Care Hospital to derive data using processing methods such as shielding personal sensitive information.

### Grouping criteria

Inclusion Criteria: (1) age 18–45 years old; (2) no smoking and alcohol addiction; (3) pregnant women with regular maternity examination and delivery in Jinan Maternal and Child Health Hospital from January 2016 to June 2022; (4) “discharge diagnosis” was GH or PE. Exclusion Criteria: (1) “discharge diagnosis” were pregnancy-associated chronic hypertension and chronic hypertension with superimposed preeclampsia; (2) secondary hypertension (such as Cushing syndrome, primary aldosteronism, pheochromocytoma, etc.); (3) severe heart failure, liver failure or kidney failure; (4) chronic kidney disease or renal angiopathy; (5) a new serious infection; (6) tumor. Group requirements: (1) GH group: systolic blood pressure (SBP) of 140 mmHg and/or diastolic blood pressure (DBP) of 90 mmHg were initially noted after 20 weeks of pregnancy in the absence of features of preeclampsia. (2) PE group: SBP of 140 mmHg and/or DBP of 90 mmHg following 20 weeks of gestation, linked to atypical urine protein (+) or any organ or system involvement (such as acute kidney injury, liver involvement, hematological complications, uteroplacental dysfunction) (18).

Sample size calculation: based on the principle (19) that the sample size should be at least 10–20 times the number of independent variables, 19 variables were included in this study: age, height, weight, BMI, BP, HR, UA, BUN, TBIL, TBA, FFA, DBIL, ALP, Scr, cystatin C, GLU, SOD, Na+, and K+. With an estimated lost-to-follow-up rate of 10%, the total sample size required would be 211–422 patients. Finally, 1,548 subjects who met the inclusion criteria were included.

### General data collection

The age, height and weight of GH and PE patients were extracted through the medical record system, and the BMI was calculated according to BMI = weight/(height^2^). Patients were seated for 10 min, and their BP and HR were measured 3 consecutive times, using a HEM 7011 blood pressure monitor (Omron Healthcare, Kyoto, Japan). Baseline BP was the mean of 3 beats and baseline HR as well.

### Laboratory methods

After 8 h of fasting, venous blood was collected in the morning from all participants. Liver function, renal function and GLU were detected by the enzymatic endpoint colorimetric method using Roche Cobas 8000 biochemical kit.

### Statistical methods

SPSS statistical software (version 25.0; IBM Corp, Armonk, NY, United States) was used for the statistical analysis. Normally distributed continuous data were reported as mean ± standard deviation (SD) and compared using an independent *t*-test. If the data did not conform to the normal distribution, the median (M) and quartile (P25, P75) are used to describe the data, and we compared those indicators between the 2 groups through the Mann–Whitney *U*-test. A difference was considered statistically significant if the *P* < 0.05. Categorical data were presented as examples and percentages using the chi-square test. Predictors influencing GH progression to PE were analyzed using whether PE occurred as the dependent variable, significantly different variables derived from the *T*-test and *U*-test were analyzed by univariable logistics. Finally, the risk factors identified and protective factors in the univariate analysis as independent variables were put into multivariate models. The diagnostic impact of each pertinent indicator was assessed using the receiver operating characteristic (ROC) curves. It was decided to create a visual Nomograms representation of the logistic. Each variable's score value was equal to the integer value of the absolute value of the variable as determined by the logistic regression prediction model's regression coefficient value. A streamlined scoring method was created after calculating the score for each variable. The sum of the various variable scores was used to do the hazard stratification.

## Results

### Comparison of general information

A total of 1,548 patients were included in this study, of whom 649 and 899 were in the GH and PE groups, respectively. The 1,548 patients were randomly divided at a 7:3 ratio into the training set (*n* = 1,083) and validation set (*n* = 465). In the 1,083 patients in the training set, 454 and 629 were in the GH and PE groups, respectively.

The general data of the 1,083 cases were matched between the 2 groups. There was no significant difference in age [34 (30, 38), 35 (31, 39), *P* = 0.14] between the 2 groups. SBP [136 (128, 144), 149 (138, 160), *P* < 0.01] mmHg, DBP [90 (82, 95), 98 (89, 106), *P* < 0.01] in the PE group were higher than GH group, while the HR [92 (85, 100), 86 (77, 95), *P* < 0.01], BMI [30.90 (28.10, 33.83), 30.49 (28.04, 33.20), *P* < 0.01] in the PE group were lower than GH group, the difference was statistically significant (Table 1).

**Table 1:** Comparison of baseline indicators between GH group and PE group.

Item	Overall (*n* = 1,083)	GH group (*n* = 454)	PE group (*n* = 629)	*P*-value
Age [year, M(*P*_25_, *P*_75_)]	34 (30, 38)	34 (30, 38)	35 (31, 39)	0.14
SBP [mmHg, M(*P*_25_, *P*_75_)]	143 (134, 155)	136 (128, 144)	149 (138, 160)*	<0.01
DBP [mmHg, M(*P*_25_, *P*_75_)]	94 (86, 103)	90 (82, 95)	98 (89, 106)*	<0.01
HR [beats/min, M(*P*_25_, *P*_75_)]	88 (81, 98)	92 (85, 100)	86 (77, 95)*	<0.01
BMI [kg/m^2^, M(*P*_25_, *P*_75_)]	30.50 (28.60, 33.40)	30.90 (28.10, 33.83)	30.49 (28.04, 33.20)*	<0.01

GH, gestation hypertension; PE, preeclampsia; SBP, systolic blood pressure; DBP, diastolic blood pressure; HR, heart rate; BMI, body mass index.

* *P* < 0.01.

### Comparison of general biochemical indicators

The main general biochemical indicators included in this study were mainly UA, BUN, TBIL, TBA, DBIL, ALP, Scr, cystatin C, GLU, SOD, Na +, K+ and UA/SOD. The T-test was used to compare the general data of the 2 groups in the 1,083 training set, results showed that TBIL [5.10 (3.63, 7.00), 6.25 (4.60, 8.28), *P* < 0.01], DBIL [2.10 (1.20, 2.90), 2.60 (2.10, 3.30), *P* < 0.01], ALP [106.00 (60.25, 150.00), 136.00 (106.50, 174.50), *P* < 0.01], GLU [4.20 (3.20, 5.00), 4.80 (4.30, 5.70), *P* < 0.01], SOD [119.30 (84.25, 140.88), 163.00 (144.25, 178.55), *P* < 0.01] in the PE group were lower than GH group. However, in the PE group, BUN [5.70 (4.20, 9.50), 3.50 (2.90, 4.10), *P* < 0.01], TBA [2.90 (1.50, 5.30), 2.80 (1.80, 4.50), *P* = 0.01], Scr [63.00 (54.00, 79.00), 44.00 (38.00, 51.00), *P* < 0.01], cystatin C [1.33 (1.09, 2.08), 1.00 (0.85, 1.19), *P* < 0.01], K+ [4.38 (4.07, 5.09), 4.01 (3.79, 4.23), *P* < 0.01], UA/SOD [3.58 (2.97, 4.21), 1.89 (1.50, 2.34), *P* < 0.01] were higher than GH group, the difference was statistically significant. The indicators UA (426.01 ± 83.43, 308.70 ± 85.64, *P* > 0.05), FFA [0.59 (0.39, 0.97), 0.48 (0.34, 0.71), *P* > 0.05], Na+ [138.00 (136.00, 140.00), 138.00 (136.00, 139.00), *P* > 0.05] were not statistically significant (Table 2).

**Table 2:** Comparison of laboratory indicators between GH group and PE group.

Item	GH group (*n* = 454)	PE group (*n* = 629)	*P*-value
UA (µmol/L)	308.70 ± 85.64	426.01 ± 83.43	0.81
BUN [mmol/L, M(*P*_25_, *P*_75_)]	3.50 (2.90, 4.10)	5.70 (4.20, 9.50)*	<0.01
TBIL [mmol/L, M(*P*_25_, *P*_75_)]	6.25 (4.60, 8.28)	5.10 (3.63, 7.00)*	<0.01
TBA [µmol/L, M(*P*_25_, *P*_75_)]	2.80 (1.80, 4.50)	2.90 (1.50, 5.30)**	0.01
FFA [mmol/L, M(*P*_25_, *P*_75_)]	0.48 (0.34, 0.71)	0.59 (0.39, 0.97)	0.39
DBIL [mmol/L, M(*P*_25_, *P*_75_)]	2.60 (2.10, 3.30)	2.10 (1.20, 2.90)*	<0.01
ALP [U/L, M(*P*_25_, *P*_75_)]	136.00 (106.50, 174.50)	106.00 (60.25, 150.00)*	<0.01
Scr [µmol/L, M(*P*_25_, *P*_75_)]	44.00 (38.00, 51.00)	63.00 (54.00, 79.00)*	<0.01
Cys-C [mg/L, M(*P*_25_, *P*_75_)]	1.00 (0.85, 1.19)	1.33 (1.09, 2.08)*	<0.01
Glu [mmol/L, M(*P*_25_, *P*_75_)]	4.80 (4.30, 5.70)	4.20 (3.20,5.00)*	<0.01
SOD [U/L, M(*P*_25_, *P*_75_)]	163.00 (144.25, 178.55)	119.30 (84.25, 140.88)*	<0.01
Na+ [mmol/L, M(*P*_25_, *P*_75_)]	138.00 (136.00, 139.00)	138.00 (136.00, 140.00)	0.42
K+ [mmol/L, M(*P*_25_, *P*_75_)]	4.01 (3.79, 4.23)	4.38 (4.07, 5.09)*	<0.01
UA/SOD [µmol/U, M(*P*_25_, *P*_75_)]	1.89 (1.50, 2.34)	3.58 (2.97, 4.21)*	<0.01

GH, gestation hypertension; PE, preeclampsia; UA, uric acid; BUN, urea nitrogen; TBIL, total bilirubin; TBA, total bile acid; FFA, free fatty acid; DBIL, direct bilirubin; ALP, alkaline phosphatase; Scr, serum creatinine; Cys-C, cystatin C; Glu, glucose; SOD, superoxide dismutase; Na+, natrium; K+, potassium.

* *P* < 0.01.

** *P* < 0.05.

### Univariate analysis

**Figure 1:**
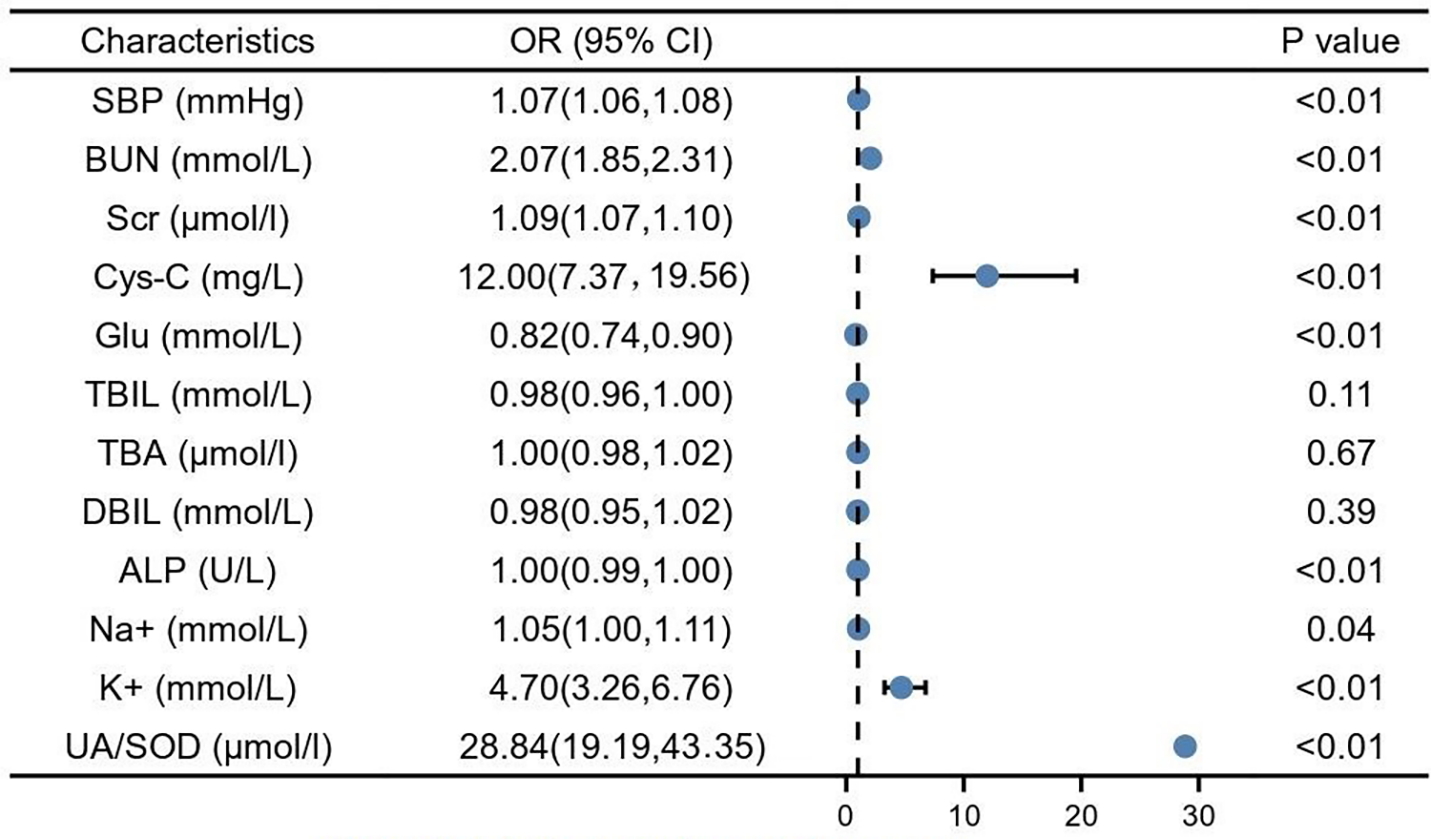
Single factor logistic analysis. SBP, systolic blood pressure; BUN, blood urea nitrogen; Scr, serum creatinine; Cys-C, cystain C; Glu, glucose; TBIL, total bilirubin; TBA, total bile acid; DBIL, direct bilirubin; ALP, alkaline phosphatase; Na+, natrium; K+, potassium; UA, blood uric acid; SOD, superoxide dismutase.

By univariate logistics analysis of indicators with significant differences derived from the *T*-test and *U*-test, results showed that SBP (1.07, 95% CI: 1.06–1.08, *P* < 0.01), BUN (2.07, 95% CI: 1.85–2.30, *P* < 0.01), Scr (1.09, 95% CI: 1.07–1.10, *P* < 0.01), cystatin C (12.00, 95% CI: 7.37–19.56, *P* < 0.01), ALP (1.00, 95% CI: 0.99–1.00, *P* < 0.01), Na+ (1.05, 95% CI: 1.00–1.11, *P* < 0.05), K+ (4.70, 95% CI: 3.26–6.76, *P* < 0.01), UA/SOD (28.84, 95% CI: 19.19–43.35, *P* < 0.01) were risk factors for developing GH to PE. However, elevated GLU seems to be a protective factor (0.82, 95% CI: 0.74–0.90, *P* < 0.01) for the development of GH to PE (Figure 1).

### Multivariate analysis

**Figure 2:**
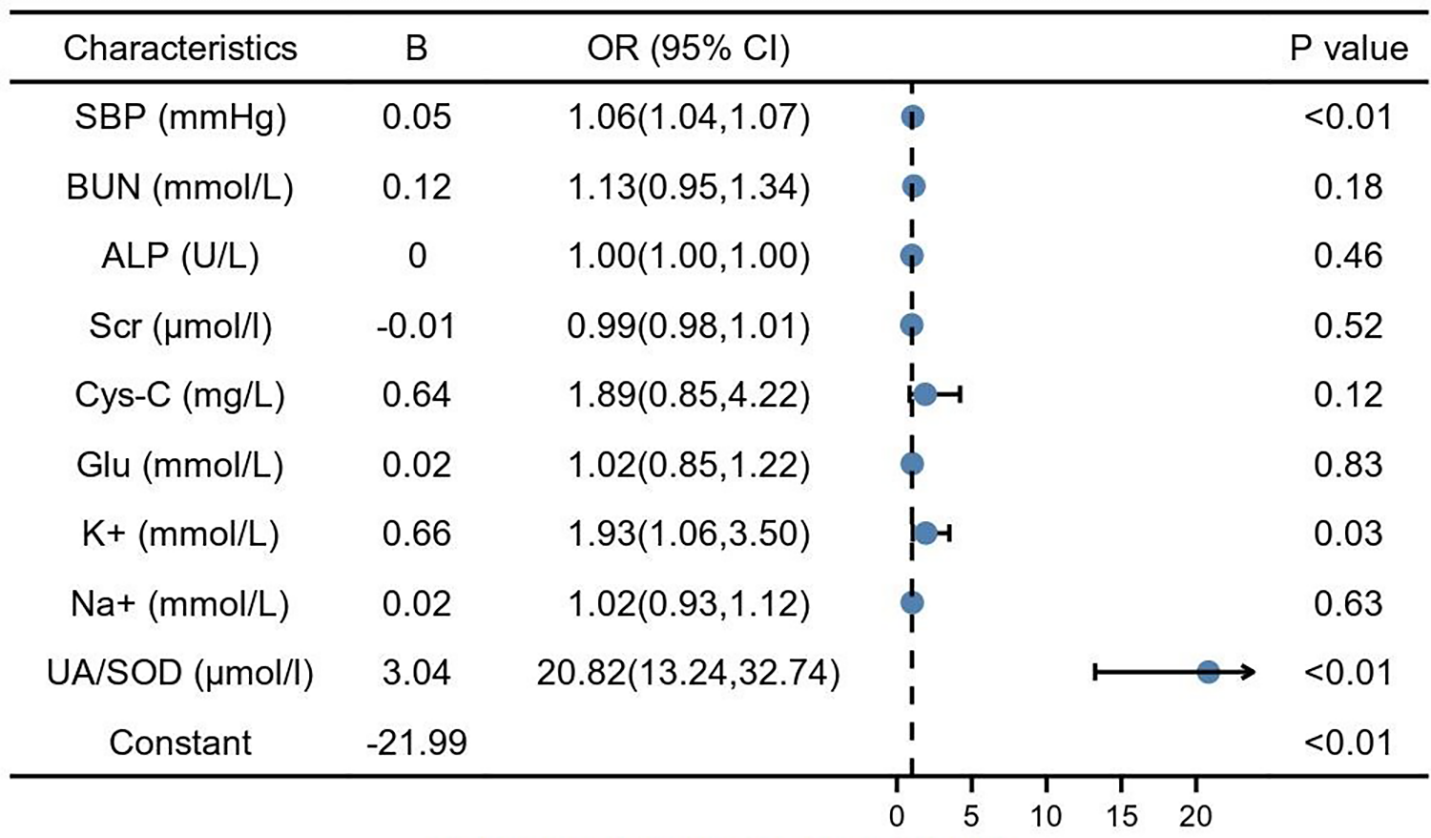
Multiple factor logistic analysis. SBP, systolic blood pressure; BUN, blood urea nitrogen; Scr, serum creatinine; Cys-C, cystain C; Glu, glucose; TBIL, total bilirubin; TBA, total bile acid; DBIL, direct bilirubin; ALP, alkaline phosphatase; Na+, natrium; K+, potassium; UA, blood uric acid; SOD, superoxide dismutase.

Predictors influencing GH progression to PE were analyzed using whether PE occurred as the dependent variable and the risk factors identified and protective factors in the univariate analysis as independent variables. SBP (1.06, 95% CI: 1.04–1.07, *P* < 0.01) and serum potassium (1.93, 95% CI: 1.06–3.50, *P* < 0.01), UA/SOD (20.82, 95% CI: 13.24–32.70, *P* < 0.01) were found to be independent risk factors for the development of GH to PE (Figure 2).

### Nomogram and validation

**Figure 3:**
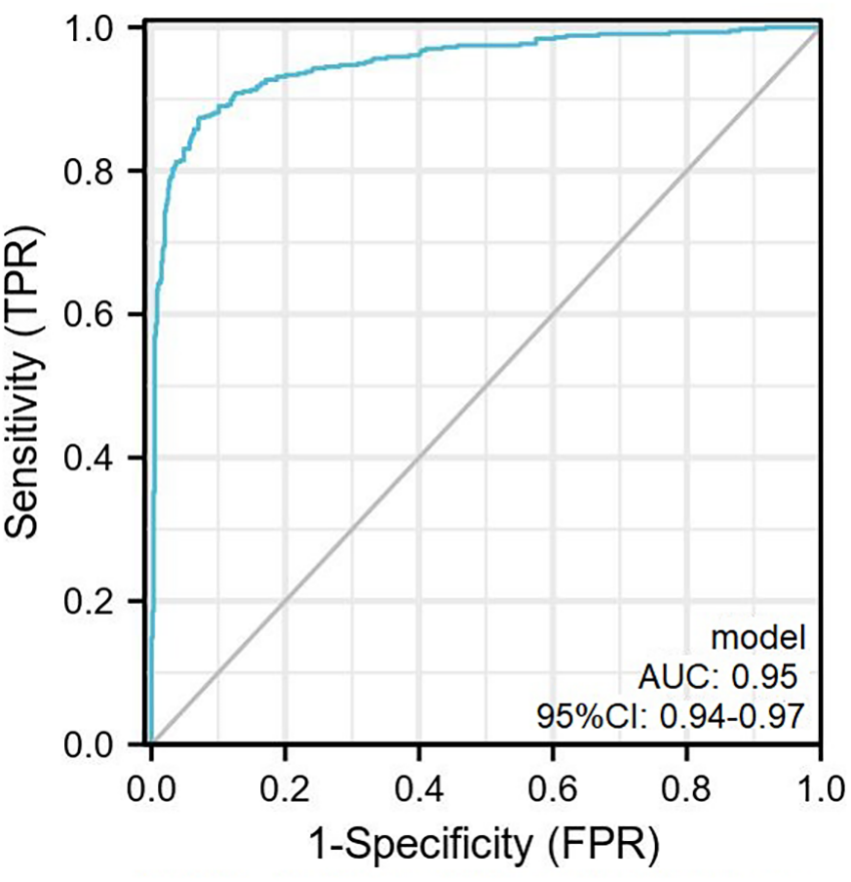
The ROC curve plotted according to the training set. The AUC of the predictive model was 0.95 (95% CI, 0.94–0.97) with sensitivity and specificity of 87.4% and 92.8% in the training set. ROC, receiver operator characteristic curve.

According to the results of the multivariate analysis, these 3 factors were included in the regression prediction model, and the calculation formula was: *Y* = −21.99 + 0.05 × (SBP) + 0.66 × (serum potassium) + 3.04 × (UA/SOD). The prediction probability of the model was used to plot a receiver operating characteristic (ROC) curve and the area under the curve was 0.95 (95% CI: 0.94–0.97), sensitivity was 87.4%, specificity was 92.8%, and maximum Youden index was 0.80 (Figure 3, Table 3).

**Table 3:** The ROC curve characteristics.

Predict variable	Cut-off	Sensitivity	Specificity	PPV	NPV	Youden index
Model	−0.07	87.4%	92.8%	89.9%	91.0%	0.80

ROC, receiver operator characteristic curve; PPV, positive predictive value; NPV, negative predictive value.

**Figure 4:**
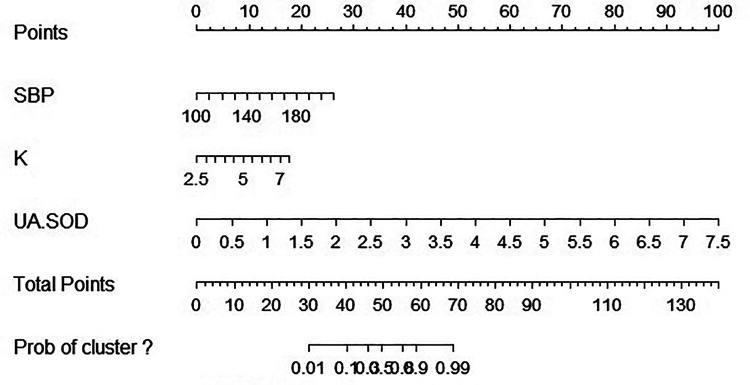
Nomographic chart. SBP, systolic blood pressure; K, potassium; UA, blood uric acid; SOD, superoxide dismutase.

In this study, nomogram was constructed and 3 independent risk factors included for visualization of the logistic model. In the nomogram, UA/SOD was the greatest predictor of PE (100 points), followed by SBP (29 points), and serum potassium (19 points) (Figure 4).

**Figure 5:**
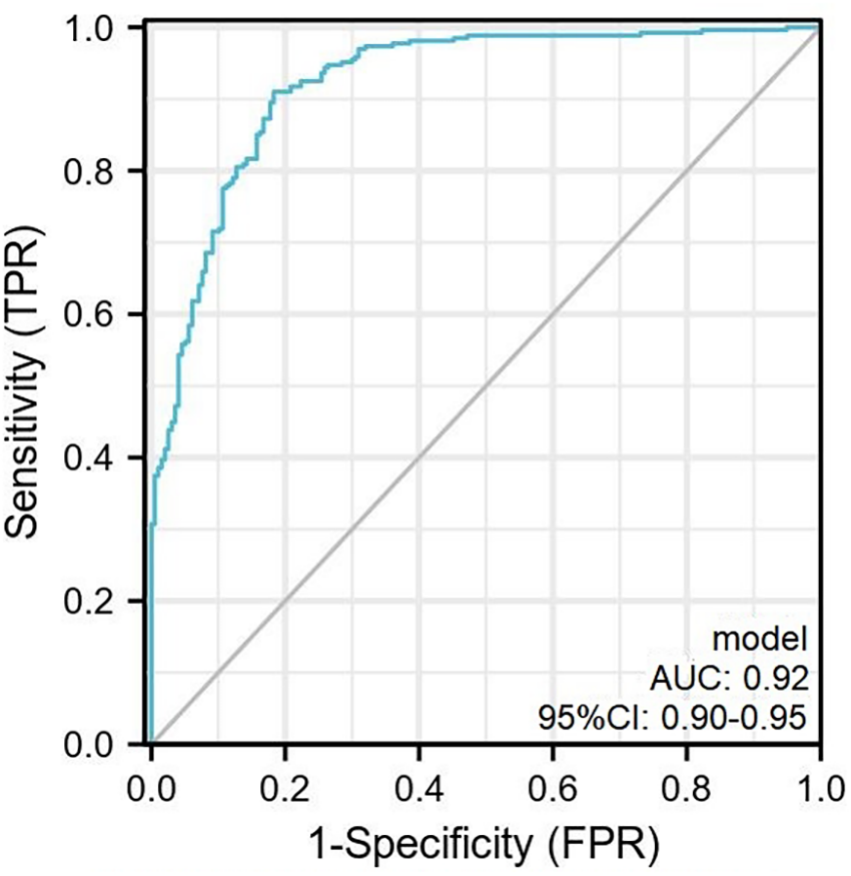
The ROC curve plotted according to the validation set. The AUC of the predictive model was 0.92 (95% CI: 0.90–0.95) with sensitivity and specificity of 91.0% and 81.7% in the validation set. ROC, receiver operator characteristic curve.

**Figure 6:**
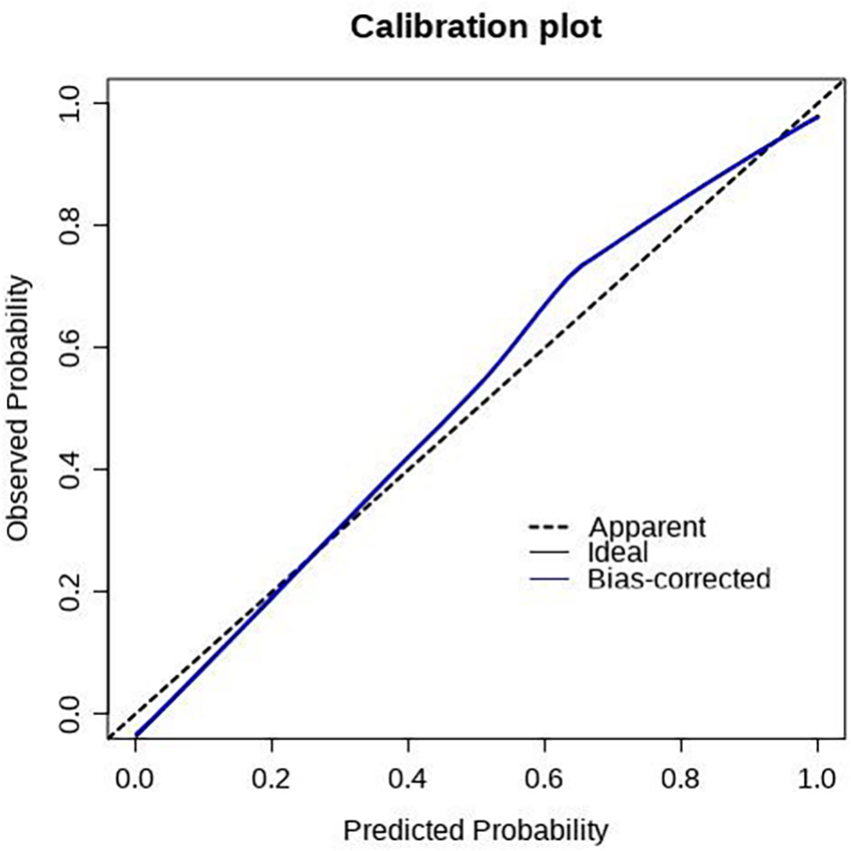
Calibration plot of the training set. The predictive models had good calibration with a mean error of 0.03 in the training set.

**Figure 7:**
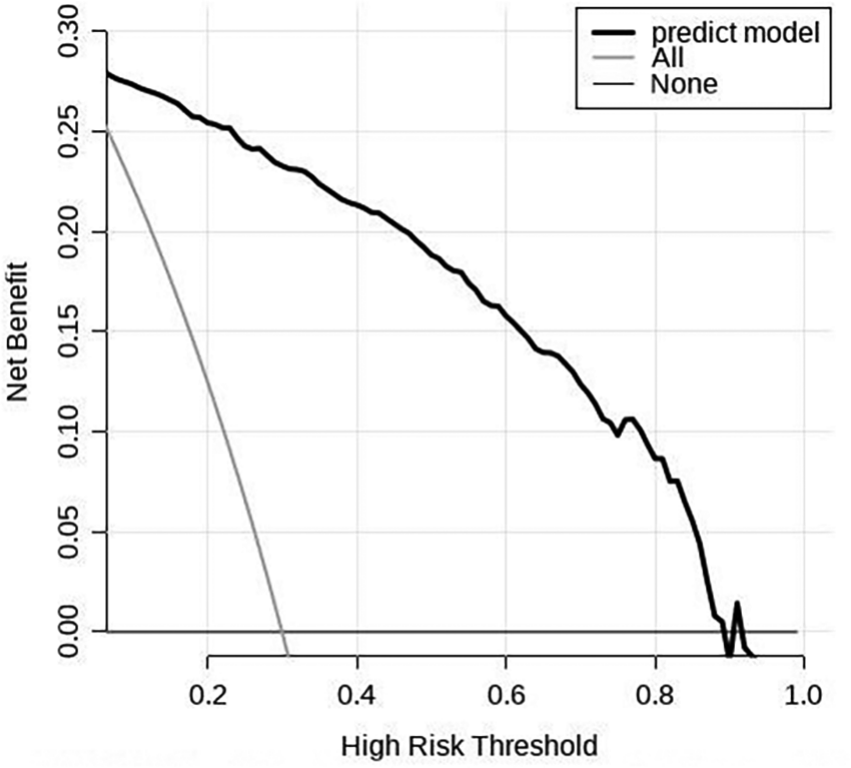
Clinical decision analysis curve of the training set. The DCA curve showed that using the model to predict the development of GH to PE produced more benefit when the probability of PE between 0.01 and 0.91.

The scoring system was used to plot the ROC curve for the validation set. The area under the curve was 0.92 (95% CI: 0.90–0.95). The maximum Youden index was 0.73, sensitivity was 91.0%, and specificity was 81.7% (Figure 5, Table 4). The calibration analysis curve found that the model had good accuracy with a mean absolute error of 0.03 (Figure 6). The clinical decision analysis curve showed that the model showed a positive benefit when the threshold was 0.01–0.91 (Figure 7).

**Table 4:** The ROC curve characteristics.

Predict variable	Cut-off	Sensitivity	Specificity	PPV	NPV	Youden index
Model	−0.15	91.0%	81.7%	87.1%	87.0%	0.73

ROC, receiver operator characteristic curve; PPV, positive predictive value; NPV, negative predictive value.

## Discussion

PE is a pregnancy-specific disease that can cause abnormal heart, brain, liver, and kidney function, which is among the major causes of maternal and peripartum infant death (20). Our previous study (21) showed that the mean gestational age when blood pressure starts to increase in PE patients was 32.54 weeks and the mean gestational age at pregnancy termination was 33.92 weeks. This shows that PE progression occurs rapidly in most patients and only 7–10 days pass from the start of blood pressure elevation to uncontrollable disease and pregnancy termination. The mean birth weight of infants from PE patients is only 2,340 g. Doctors have to use dexamethasone to promote fetal lung maturation to reduce neonatal mortality. This treatment takes only 36 h. There is an urgent need for a prediction method for early PE detection in clinical practice, thereby allowing early PE intervention treatment to ensure maternal and infant safety.

This study was based on our previous study. The sample size was expanded, and disease coverage was increased. A real-world big data analysis was employed, and 1,548 GH and PE patients were selected. We focused on clinical predictors of GH progression to PE. This study mainly examined whether oxidative stress can effectively predict PE. Its results showed that UA/SOD can predict the progression of GH to PE. Compared with UA or SOD alone for predicting PE, the use of oxidant to antioxidant ratio can better reflect the *in vivo* oxidative stress status and has higher diagnostic value. To further validate the accuracy of our study results, we assigned weights of 100, 29, and 19 to UA/SOD, SBP, and serum potassium, respectively, based on multivariate logistic regression analysis. Thereafter, we plotted the ROC curve. The area under the curve was 0.92, sensitivity was 91.0%, and specificity was 81.7%. The results showed that our model had extremely good prediction performance. This means that if a clinician discovers a GH patient, she can measure the UA/SOD to predict the probability of progression to PE. This can guide clinicians to strengthen pregnancy monitoring of patients at high risk for developing PE. Once disease progression is identified, early pressure-lowering and fetal lung maturation treatments can be administered to improve maternal and infant prognosis. UA/SOD has extremely important significance in predicting the progression of GH to PE.

There are currently many markers for PE prediction in clinical practice, such as platelets, serum calcium, placental growth factor, and endothelin. However, none have high specificity. Jhee et al. (22) screened 11,006 pregnant women and used SBP, BUN, Scr, platelet count, white blood cell count, serum calcium, and serum magnesium levels to construct a regression equation based on the calculation results. However, the markers involved are complex and unsuitable for large-scale clinical screenings. Some studies also used hypoxia inducible factor-1a (23), vascular endothelial growth factor (24), signal transducer and activator of transcription 3 (25), and lipoprotein-associated phospholipase A2 (26) to predict PE, but the cost is high and its use not suitable for promotion. Thus, there is an urgent need for a more accurate, simpler, and cheaper clinical marker to predict PE.

A prospective single-center cohort study of GH pregnant women found that UA level has extremely important significance in predicting the progression of GH to PE, with an area under the ROC curve of 0.85 and sensitivity of 90.7% (27). The results of that study were similar to our study results. However, that study only had a positive predictive value of 34% and negative predictive value of 97.7%, but our study had a positive predictive value of 89.9% and negative predictive value of 91.0%. Based on the above studies, this study was the first to find that UA/SOD can better predict GH progression to PE than UA or SOD alone. This marker is inexpensive, is easily obtained, and has high specificity, and it is a preferred item for large-scale clinical screening. This study also found that SBP and serum potassium are important risk factors for GH progression to PE.

A higher BP usually causes target organ damage, and the most common target organ is the kidneys. The definition of PE is hypertension accompanied by heart, brain, liver, and kidney involvement of placental-fetal involvement after gestational week 20. Therefore, BP has an extremely important correlation with the occurrence of PE (18). Moreover, this study also found that the mean serum population level of the enrolled population was within the normal range and serum potassium was slightly higher in the PE group than in the GH group. Our previous study (21) found that serum calcium is lower in PE than GH pregnant women. Potassium and calcium ions are both cations that competitively bind to cation channels. An elevated serum potassium will inhibit calcium absorption, resulting in decreased serum calcium, a risk factor for PE. This was similar to the results of a study by Hofmeyr et al. (28). Another study (29) showed that elevated serum potassium is associated with pregnancy-associated acute kidney injury; PE is the most important cause. This indirectly shows that elevated serum potassium can mutually affect PE.

Although we enrolled 1,548 patients in this study, we did not compare them with healthy pregnant women. In subsequent studies, we will continue to expand the sample size and study population and use real-world big data studies to validate the clinical markers of progression to PE in healthy pregnant women to identify earlier predictors. Furthermore, this was a single-center study. We will use its results to conduct a multicenter study to further validate its predictive performance.

## Conclusion

In conclusion, SBP, serum potassium and UA/SOD were independent risk factors for the development of GH to PE. The risk factor prediction model and scoring system established by these 3 items have high sensitivity and specificity, which can provide good estimates to the risk stratification of PE. UA/SOD can predict progression of GH to PE.

## Data Availability

The raw data supporting the conclusions of this article will be made available by the authors, without undue reservation.
